# Bush-Whacking

**Published:** 1889-08

**Authors:** 


F. E. DANIEL, M. D., EDITOR AND PUBLISHER.

THIS JOURNAL, by unanimous vote of the members, was made the Official Organ of the Austin District Medical Society.

COLLABORATORS:

*Dr. R. M. Swearingen, Austin.*
*Prof. B. E. Hadra, M. D., Galveston.*
*Prof. Geo. Cupples, M. D., San Antonio.*
*Dr. T. C. Osborn, Cleburne, Texas.*
*Dr E. J. Doering, Chicago.*
*Dr. E. J. Beall, Fort Worth.*
*Dr Odo Betz, Germany.*
*Dr. J. W. McLaughlin, Austin.*
*Dr. T. J. Tyner, Austin.*
*Prof. J. F. Y. Paine, M. D., Galveston.*
*Dr. R. H. L. Bibb, Mexico.*
*Dr. T. J. Bennett. Austin.*
*Dr. Bat Smith, Wharton.*
*Dr. E. Meierhof, New York.*
*Prof. W. B. Rogers, M. D., Memphis, Tenn.*

### BUSH-WHACKING.

There is published in Chicago a so-called *Medical Standard* (a very low "standard" truly,) an anonymous sheet which, in the brief space of its existence has, by a persistent course of misrepresentation, and distortion of the utterances of its contemporaries, continually snarling and snapping, incurred the contempt of the entire reputable portion of the medical press. It is a kind of journalistic *Ishmael*, whose hand seems against everybody.

This sheet sometime back assaulted the *Journal of the American Medical Association*, attacking Prof. Davis under an *incog.*, attributing words to him which he never uttered, and criticising them in a shameful manner;—doubtless hoping to provoke the great man to notice,—even to kick him;—but Dr. Davis cannot condescend even to kick everything that snaps at his heels.

The latest piece of impudence and downright falsification on the part of this no name editor occurs in the July number of the *Standard.*

In our last issue we pointed out the fact that a Chicago Journal, edited by representatives of the Chicago Post Graduate Medical College, is carrying in its pages the advertisement of a notorious nostrum and "patent" medicine. The *Standard* copies this, and says,—referring to us,—"The chief editor of that "organ," having recently been the servile tool of local politicians in a disreputable plot against a brother physician, we hardly expected a high ethical tone in a journal edited by him."

(Hello, have we unwittingly stepped on the "Unknown's" toes? Well, things that crawl in the dark and sting people, must expect to get stepped on.)

The above is, we suppose, an allusion to the trial (and conviction) of Superintendent Dorset, of the Austin, Texas, Lunatic Asylum, on charges brought by Dr. Johnson, an ex-employe. With this we had no more to do than the man in the moon; nor had any "local politicians" any hand in it, so far as we are aware.

In the exercise of our privilege as a journalist we criticised the appointment as superintendent, of one whom we knew to be inexperienced, (and therefore incompetent;) and as a matter of general interest to the profession we published the charge, the trial and the verdict, which we unquestionably had a right to do. But our only connection with the affair, one way or another, was, while present at the trial on a written invitation from Dr. Dorset, we were, unexpectedly, requested to testify to a conversation had with Dr. Dorset relative to the appointment of his assistants. This we did.

It was our intention to say, only, in reply to the above libelous charge, that being *anonymous*, it is as cowardly and contemptible as it is maliciously false; but we see such evidence in the last issue of the "Standard" that the kettle has called the pot names, that we cannot refrain from mentioning it. The "Standard" has, all along, been on the side of, and defended the "boodle" appointees in the Cook County Lunatic Asylum, and now that the superintendent has been removed (on charges, we believe) and a successor appointed, this conscienceless and concealed editor, whoever he (she or it) may be,—instigated, no doubt, by the "boodle" politicians,—attacks him, (under cover, of course,) accusing him of inexperience and incompetency, and even charges that he has *killed his patients* to make room for new cases; and this in the column adjoining the one, and in the same issue, in which we are charged with being in a plot against a professional brother! Kettle and pot. The "Standard" has thus shown itself to be the "servile tool of local politicians, in a disreputable plot against a brother physician;" the very charge it makes so maliciously against us can, with truth, be brought to its own door, and this hidden fellow stands convicted by his (her or its) own utterances.

Doubtless this bushwhacker, who is ashamed of his (her or its) name, if he (she or it) have one, feels secure in his concealment; but those who hire and hide him, are warned to muzzle him, or they may find themselves party to a suit for libel; while if not the fate of Ananias befall him, the righteous wrath and indignation of some outraged gentleman will overtake him in his hiding, and a horsewhip will teach him to keep a civil, at least a truthful tongue in his head.

So long as this journalistic pirate sails under no colors, the press should not recognize it as legitimate, and we apologize to our readers for having devoted so much space to so unworthy an object.

